# Silent Intruder: Unusual Presentation of Neurocysticercosis in an HIV-Infected Patient from the Far Northern Brazilian Amazon

**DOI:** 10.3390/medicina60030489

**Published:** 2024-03-16

**Authors:** Luis E. B. Galan, Letícia R. M. Gerolin, Tháilla J. M. Carvalho, Eloise T. M. Filardi, Dafnin L. S. Ramos, Domingos S. M. Dantas, Roberto C. C. Carbonell, Felipe A. Cerni, Manuela B. Pucca

**Affiliations:** 1 Medical School, Federal University of Roraima, Boa Vista 69310-000, Roraima, Brazil; luis.bermejo@ufrr.br (L.E.B.G.); marajo.leticia@gmail.com (L.R.M.G.); thailla_jasminie@hotmail.com (T.J.M.C.); dafnin.lima@gmail.com (D.L.S.R.); rcccarbonell@yahoo.es (R.C.C.C.); 2 Graduate Program in Bioscience and Biotechnology Applied to Pharmacy, School of Pharmaceutical Sciences, São Paulo State University (UNESP), Campus Araraquara, Araraquara 19060-900, São Paulo, Brazil; e.filardi@unesp.br; 3 Programa Doutoral de Bioética da Faculdade de Medicina do Porto, 4050-290 Cidade do Porto, Portugal; saviojuazeiro@yahoo.com.br; 4 Graduate Program in Tropical Medicine (PPGMT), State University of Amazonas, Manaus 69850-000, Amazonas, Brazil; felipe_cerni@hotmail.com; 5 Department of Clinical Analysis, School of Pharmaceutical Sciences, São Paulo State University (UNESP), Campus Araraquara, Araraquara 19060-900, São Paulo, Brazil

**Keywords:** parasitic infection, neurocysticercosis, subarachnoid, extraparenchymal manifestation, Roraima, Amazon

## Abstract

Neurocysticercosis, a parasitic infection of the central nervous system (CNS), is a significant public health issue globally, including in Brazil. This article presents a case report of a 44-year-old male patient residing in the rural area of Roraima, the northernmost region of Brazil within the Amazon Forest. The patient, with chronic HIV infection, acquired the *Taenia solium* helminth, resulting in neurocysticercosis development. Remarkably, the diagnosis of neurocysticercosis was not initially apparent but emerged through meticulous analysis following a motorcycle accident. The absence of seizures, a common clinical manifestation, complicated the diagnostic process, making it an uncommon case of NCC, which may be related to co-infection. As the patient’s condition progressed, multiple complications arose, requiring additional medical attention and interventions. This case underscores the immense challenges faced by healthcare teams in managing neurocysticercosis effectively. It emphasizes the critical need for a comprehensive, multidisciplinary approach to provide optimal care for such complex cases. The study’s findings underscore the importance of raising awareness and implementing improved strategies for tackling neurocysticercosis, particularly in regions where it remains a prevalent concern.

## 1. Introduction

Neurocysticercosis (NCC) is an endemic parasitic disease in many countries in Africa, Asia, and Latin America [1] and is associated with poor living conditions and low socioeconomic status, posing a significant public health problem [2]. This condition results from the infection of *Taenia solium* cysticerci in the central nervous system [3]. In 2010, cysticercosis was added to the World Health Organization’s (WHO) list of neglected tropical diseases [4]. Estimates reveal that over 50 million people worldwide were infected with the taeniasis–cysticercosis complex in 2001 [2]. However, numerous cases remain unreported, making it difficult to obtain an accurate assessment of the overall disease burden.

In Brazil, a total of 1829 deaths related to NCC were registered between the years 2000 and 2011. Regarding its distribution, studies have indicated a decline in mortality rates in the southeast, south, and midwest regions, while the north and northeast regions experienced a slight increase. This trend could be attributed to the poorer economic conditions in these regions [5]. It is widely recognized that individuals in the states of São Paulo, Minas Gerais, Paraná, and Goiás are particularly susceptible to this condition. Nationally, the rural population is the most affected, with individuals between the ages of 21 and 40 being particularly susceptible. Regarding gender, NCC exhibits severe manifestations in females but is more prevalent among males, often associated with agricultural occupations [6,7].

*Taenia solium,* the helminth accountable for neurocysticercosis, involves both pigs and humans as hosts in its biological cycle [8]. Extraintestinal infection arises when individuals ingest *Taenia solium* eggs. Afterward, these eggs penetrate the gastric wall, enter the bloodstream, and spread throughout the body [4]. Notably, they can infiltrate the central nervous system, where they may develop into cysticerci, leading to the onset of neurocysticercosis.

The clinical manifestations of neurocysticercosis are diverse. However, the majority of cases are asymptomatic, due to factors inherent to the infected person and the parasite highlighted below, which influence the clinical manifestations of NCC. The cysts trigger inflammatory responses, which consequently generate symptoms, when they come into contact with defense cells. Immunocompetent people can have an asymptomatic inflammatory reaction when they host viable forms of cysticerci, as these parasites evade and suppress the immune system, dragging out the infectious condition for years. In the case of immunocompromised people, the immune system response affected by viral infection allows uncontrolled replication of the parasite, which leads to a long asymptomatic period [9]. When symptoms are present, they may include headache, seizures, focal deficits, intracranial hypertension, hydrocephalus, meningitis, and psychiatric alterations. It is estimated that *Taenia solium* infection is responsible for up to 30% of epilepsy cases in areas where the disease is endemic [1,4].

It is also important to highlight the involvement of neurocysticercosis with the immune response, which suggests that individuals with HIV have greater susceptibility. The HIV/AIDS epidemic began in the 1980s and remains a significant public health problem. The most common opportunistic infections of the central nervous system (CNS) resulting from this infection are toxoplasmosis, cryptococcosis, and tuberculosis [10]. However, HIV infection has also been identified as an important factor in the development of NCC [5], although there is still no clear relationship between these two pathologies [11]. HIV/AIDS promotes various alterations in the immune system, primarily related to cellular immunity and the function of CD4+ T lymphocytes [10], as well as inflammatory and toxic processes in certain viral proteins within neurons [12]. It is necessary to evaluate the possibility that the immunosuppression caused by HIV/AIDS influences *T. solium* infection, which may favor the invasion and growth of parasites in the CNS [10]. It is known that as HIV infection progresses to AIDS, there is a decrease in the number and function of helper T cells (Th), accompanied by hypergammaglobulinemia. Since these lymphocytes are below expected levels, they are not efficient in eliminating HIV-infected cells, allowing the virus to proliferate and continue causing immune depletion by attacking defense cells, CD4+ T cells [13,14]. Therefore, untreated HIV-positive patients are at higher risk of opportunistic infections [15]. The decline in immune patterns, characteristic of late-stage HIV infection, can make cysticercosis prone to remaining asymptomatic, and in these cases, it is often associated with NCC, as the clinical diagnosis becomes more challenging due to cellular alterations [16]. Further investigations are proposed to understand the role of the immune system in the relationship between the parasite and the host, as well as the potential interaction of NCC in people living with HIV/AIDS. Conducting a comprehensive investigation into potential infectious correlations among patients with similar cases, while carefully considering the individual nuances of each person, is necessary.

Several factors influence NCC manifestations, such as the number of parasites, host immune reactions, the developmental stage, and the location of the cysticerci [2]. Regarding location, neural infection can be parenchymal, affecting the brain parenchyma or sulci of the subarachnoid space, or it can be extraparenchymal, involving the subarachnoid space of cisterns, vestibular system, Sylvian fissure, or spinal cord. Parenchymal NCC is more prevalent than extraparenchymal NCC [17].

In parenchymal neurocysticercosis, the main clinical manifestation is seizures [18], resulting from inflammation caused by the cysticercus, which progresses with parenchymal irritation [19]. In extraparenchymal infection, cysticerci can cause intracranial hypertension with hemiparesis, hydrocephalus, partial seizures, and focal neurological signs due to obstruction of cerebrospinal fluid flow or a strong inflammatory reaction, and if not properly treated, it can lead to death and disability [20]. The prevalence of extraparenchymal NCC is still not well understood, but it is a condition with a poor prognosis, and the mortality rate ranges from 20% to 50% of affected patients [21].

For the diagnosis of neurocysticercosis, neuroimaging examinations such as computed tomography (CT) and magnetic resonance imaging (MRI) are necessary [4,20], with the latter being more sensitive as it allows for better recognition of parasites, unlike CT which is better at detecting calcifications [22]. Serological tests [4], as well as the analysis of cerebrospinal fluid (CSF) data, are also crucial to confirm neural infection [23,24]. Furthermore, a direct visualization of the parasite through fundoscopic examination constitutes a pathognomonic finding for the diagnosis of cysticercosis [25]. Therefore, combining these imaging methods, immunological analysis, and clinical findings confirms the diagnosis of NCC [4].

The treatment of neurocysticercosis can be conservative, involving the use of corticosteroids, anthelmintic drugs, and antiepileptic medications to combat the parasites, reduce inflammation, and minimize the recurrence of seizures, respectively. Symptomatic treatment is also provided based on each patient’s needs. Due to the greater severity of the disease, the management of extraparenchymal NCC requires increased attention and may also require neurosurgical intervention [4]. Here, we present a case report of subarachnoid neurocysticercosis in a 44-year-old patient with chronic HIV, diagnosed 18 years ago, who did not present convulsive seizures as a clinical manifestation, which is commonly observed, but developed several complications.

## 2. Case Presentation

The patient was diagnosed with chronic HIV infection 18 years ago and is on regular antiretroviral therapy, which includes lamivudine, tenofovir, atazanavir, and booster with ritonavir, maintaining viral suppression, that is, undetectable virus, and a current TCD4+ cell count of 205 cells/mm^3^. The patient’s CD4+ cell count reached a minimum value of 71.

**Figure 1:**
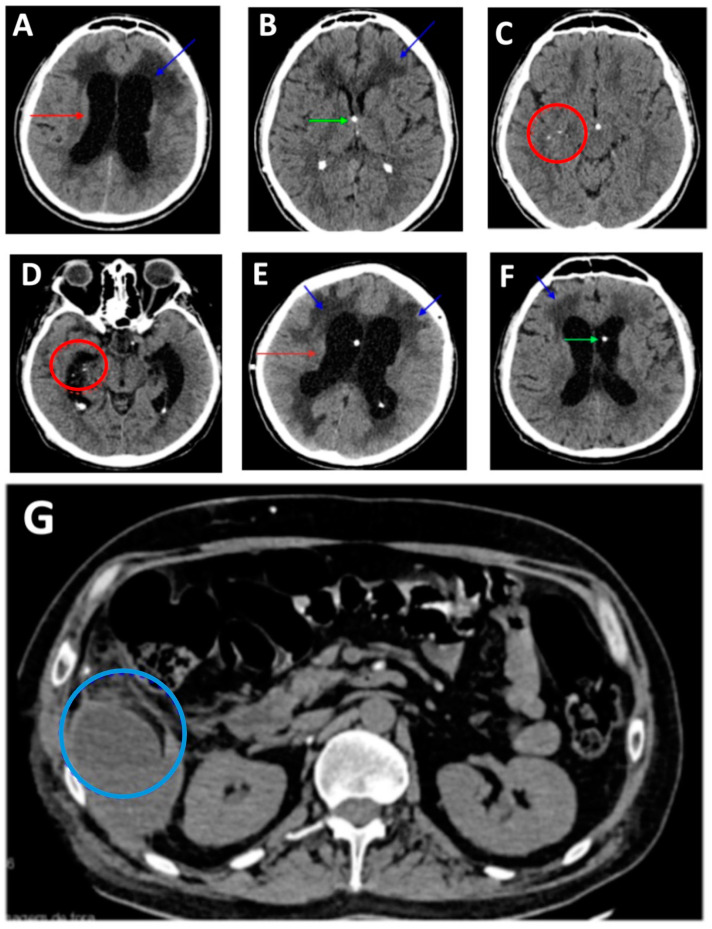
(**A**) Axial view showing the presence of hydrocephalus with ventricular dilation (red arrow) and edema (blue arrow). (**B**) Presence of edema (blue arrow) following ventriculoperitoneal shunt placement (green arrow). (**C**,**D**) Axial view showing the presence of calcifications in the subarachnoid region: red circle in both images. (**E**) Axial view demonstrating hydrocephalus with ventricular dilation (red arrow) and the presence of edema (blue arrow). (**F**) The ventriculoperitoneal shunt catheter (green arrow). (**G**) Axial view showing a subhepatic collection (blue circle).

On 20 September 2022, he was admitted to the Rubens de Souza Bento General Hospital of Roraima (HGR) due to a motorcycle accident, complaining of headache and right-sided hemiparesis. The patient reported that when leaving his motorcycle, he lost his balance and fell on a public road, progressing with the manifestation of symptoms. He stated that he had difficulty moving about 30 days before being admitted to the hospital. There is a strong suggestion that the incident, through loss of consciousness, triggered the symptoms reported for a subsequent NCC investigation, which could indicate whether or not it is a complication of the condition that was previously asymptomatic. He had no other co-morbidities, but he had a history of previous consultations for symptoms attributed to a stroke and imaging tests showed possible sequelae of old ischemic diseases due to the area of encephalomalacia/gliosis in the anterior aspect of the right striatum. Upon admission, imaging tests such as computed tomography and magnetic resonance imaging of the skull were performed, revealing supra- and infratentorial hydrocephalus with signs of edema, as well as adhesions causing loculations in the subarachnoid space (Figure 1). During the evaluation, it was found that the patient presented the triad of urinary incontinence, gait disturbance, and behavioral changes suggestive of normal-pressure hydrocephalus.

Following the diagnosis of normal-pressure hydrocephalus, an array of tests were conducted on the cerebrospinal fluid (CSF), encompassing culture and molecular assessments (Table 1 and Table 2). The CSF exhibited elevated protein levels, diminished glucose, and pleocytosis characterized by an increase in mononuclear cells. Employing a swift molecular test, *Mycobacterium tuberculosis* was examined, yielding a negative outcome, further affirmed by subsequent unyielding cultures. Likewise, fungal culture provided a negative result. The need for these tests in the diagnostic investigation was necessary to exclude other pathologies related to clinical manifestations. Integrating the patient’s clinical, radiological, and epidemiological insights and CSF findings, a direct ELISA test targeting the cysticercal antigen was performed on the cerebrospinal fluid. The ELISA assay manifested reactivity to cysticercosis. The ELISA test report was ‘reactive’, confirming the presence of the antigen in CSF and the diagnosis. Although there are various techniques available for detecting cysticercosis, immunoenzymatic techniques (ELISA) have demonstrated superior performance in diagnosis when compared to immunofluorescence (IFI) or hemagglutination (HA) techniques. ELISA is the most widely utilized method for detecting specific antibodies and circulating cysticercal antigens in samples of biological fluids, including serum, cerebrospinal fluid, or urine [22].

Concurrently considering the medical history and clinical presentation, this cumulative evidence decisively confirmed the presence of subarachnoid neurocysticercosis. Shortly after diagnosis, the patient commenced the initial cycle of therapy with albendazole and dexamethasone. Corticosteroids were initiated at the outset, followed by antiparasitic medication, which was administered over a three-week period. However, owing to elevated transaminase levels, the dosage of the antiparasitic had to be adjusted downwards. Before undergoing the ventriculoperitoneal shunt (VPS) procedure, the patient was on albendazole and dexamethasone therapy.

On October 8, ventriculoperitoneal shunting (VPS) was indicated for the resolution of hydrocephalus, resulting in significant clinical and radiological improvement. A new computed tomograph also revealed calcified parenchymal lesions (Figure 1). Identification of the appearance of the cyst on the most recent imaging was determined only after neurosurgical intervention.

During the preoperative period, the antiretroviral treatment was simplified, and the patient switched to dual therapy with dolutegravir and lamivudine. Antiparasitic treatment commenced with albendazole (400 mg orally every 8 h), coupled with the corticosteroid dexamethasone (4 mg administered intravenously every 12 h). This regimen persisted during the postoperative period and upon the patient’s return to the hospital. Additionally, a multidisciplinary treatment approach, incorporating medical specialties such as neurosurgery, psychology, and physiotherapy, was implemented, resulting in notable overall improvement, particularly in symptoms. The patient was discharged on 26 October 2022 and demonstrated the ability to walk with the assistance of orthopedic devices.

On 3 February 2023, the patient returned to HGR with worsening clinical symptoms, including recurrence of lower limb paralysis, and imaging findings of hydrocephalus related to the obstruction of the previously performed VPS (Figure 1). Therefore, a valve replacement was performed. The neurocysticercosis treatment regimen involving albendazole and dexamethasone was reinstated, albeit with a lower dosage of albendazole, attributed to hepatotoxicity resulting from polypharmacy. Patient was discharged.

On 12 March 2023, the patient returned to HGR with a febrile condition, vomiting, abdominal pain, and headache. During medical evaluation and imaging tests, a subhepatic collection on the right side was observed, and the condition worsened, requiring hospitalization for better therapeutic management of the complication. The VPS was removed, and an external ventricular drain (EVD) was placed. Throughout the patient timeframe, a series of complications emerged, including deep venous thrombosis, Candida meningitis, and cerebrospinal fluid retention. The patient requires medical monitoring due to HIV positivity. The presence of complications was monitored until March 2023. On 9 February 2023, the patient had a CD4+ count of 72 cells/mm^3^, with good adherence to antiretroviral treatment. To address these complexities, the patient received substantial antibiotic doses—specifically, sulfamethoxazole + trimethoprim at 400/80 mg, administered as two tablets orally once a day. Additionally, anticoagulants were administered, namely enoxaparin at 40 mg, administered as a 0.4 mL subcutaneous dose once daily. Interestingly, despite the depicted scenario and the evident parenchymal engagement, seizures were notably absent. Following six weeks of treatment, during which the patient exhibited noticeable clinical improvement, the corticosteroid was gradually tapered over a two-month period until the patient was discharged.

**Figure 2:**
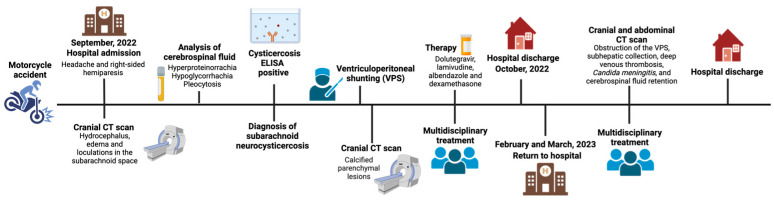
Timeline of the case of subarachnoid neurocysticercosis, illustrating the chronological sequence of events. Created with BioRender.com.

Considering all the information about different ways the illness manifested, the patient coming back to the hospital many times (Figure 2), and the patient staying in the hospital for a long time, this case was challenging for the doctors and healthcare teams.

The study was approved by the Research Ethics Committee of the Federal University of Roraima, under protocol number CAAE 68517623.6.0000.5302, and written informed consent was obtained from the patient to publish all included information.

## 3. Discussion

Neurocysticercosis is one of the main diseases caused by parasites in humans, and it is considered a public health problem [26], given that high morbidity and mortality mainly affect endemic regions [17]. The patient under investigation falls within the risk parameters for contracting the disease, not only as a rural inhabitant but also due to their occupation that entails close contact with pig farming, which is a direct source of contamination.

The patient under study sought hospital care after a motorcycle accident. On this occasion, some of the patient’s complaints were observed, such as progressive weakness in the right lower limb, headache, urinary incontinence, and mood changes, which were attributed to episodes of stroke, generating diagnostic suspicion initially and later related to NCC. The most common manifestations of NCC are seizures, but headache, hemiparesis, and ataxia may also be present [27], which supports the presented case and makes it unusual given the absence of seizures, a symptom not presented by the patient in question. It is crucial to highlight that NCC symptoms may persist for an extended period, be transient, or occur sporadically, often eluding recognition and diagnosis during medical assessments [28], mainly in subarachnoid NCC, with a pleomorphic presentation, ranging from asymptomatic cases to meningitis and hydrocephalus [29].

Among HIV-infected patients, cysticercosis has emerged as one of the various opportunistic infections capable of causing focal brain lesions, although NCC is not the most common cause in these individuals [30]. Among the identified cases of patients co-infected with NCC and HIV, the main symptom presented by the patients was seizures and they had multiple parasites located in the parenchyma. In patients with only extraparenchymal parasites, the location was predominantly the subarachnoid space [10]. This condition has garnered particular attention due to its implications for individuals with a compromised immune system, underlining the importance of early detection and appropriate management [16]. It is important to highlight that diagnosing this disease in patients living with HIV can be challenging due to the prevalence of other causes of brain lesions, such as toxoplasmosis and tuberculosis [31], as well as the reduced sensitivity of diagnostic tests in these individuals [32], often accompanied by immune parameter disorders [16]. In the case presented here, the diagnosis was suspected and later confirmed based on a combination of clinical, epidemiological, and imaging findings, primarily due to the disease’s atypical presentation.

Much like the impact of *T. solium* helminth infection, HIV infection also induces immunological changes in the central nervous system (CNS). Recognizing these concurrent effects is pivotal, as it unveils the complex interplay between infectious agents and immune responses within the CNS. This understanding contributes to advancements in medical interventions and treatments for both conditions [11]. When juxtaposing the two infections and their implications on the immune system, it is essential to acknowledge that the immunosuppression caused by HIV can significantly affect responses to *T. solium* infection [10], potentially contributing to the severity of neuroinfection development [33]. The pathophysiological interactions between HIV/AIDS and NCC are still poorly understood, but the viral infection allows viral replication of the parasite [9], in addition to the fact that asymptomatic NCC can develop into an immune reconstitution inflammatory syndrome (IRIS), becoming symptomatic in the presence of antiretroviral therapy [34]

In NCC, a diverse range of cells secrete both Th1 and Th2 cytokines, which play a crucial role in chronic immunity. Parasites can trigger the production of Th2 cytokines to control the parasite’s growth [35,36], while the death of the cysticercus is associated with Th1 production [36]. The brain aims to minimize inflammation to preserve its integrity and function, with the immune response primarily involving Th1 and Th2 responses, particularly the latter, which protects the brain tissue and activates macrophages and plasma cells [35]. The presence of symptoms is primarily linked to the host’s inflammatory response, and in patients with HIV/NCC co-infection, a less effective immune and inflammatory response may lead to a prolonged lifespan of the cysticercus, resulting in asymptomatic patients [27,36]. HIV–NCC co-infection can influence a scenario, as both pathologies affect the immune system, which can favor the growth of the parasite and especially radiological severity [10].

It is suggested that the clinical presentation might also be influenced by the number of CD4+ T lymphocytes. When these cells are spared, the appearance of symptoms becomes more likely, as the manifestation of neurocysticercosis symptoms relies on the host’s inflammatory response [36]. In patients with active stages of HIV, for instance, epileptic seizures are more common in those with higher CD4+ T lymphocyte counts [34], highlighting the impact of immune suppression on the patient’s clinical condition. This observation is particularly relevant in the presented case, where the patient has been living with chronic HIV infection for 18 years, potentially contributing to the development of neurocysticercosis.

Indeed, the patient in the study showed fluctuations in CD4+ T lymphocyte counts in the last three exams, with values of 205 cells/mm^3^ in September 2022, 71 cells/mm^3^ in November 2022, and 123 cells/mm^3^ in April 2023, which along with periodic use of corticotherapy, could potentially impact the patient’s clinical presentation, since monitoring TCD4+ and TCD8+ lymphocytes and viral load is necessary, as the decrease in lymphocytes puts the patient in a state of immunological suppression, which makes them susceptible to various infections [37]. Among all the values presented by the patient, the nadir of CD4 was 71. The gradual drop in CD4 lymphocytes may be related to the clinical latency of HIV/AIDS. As the infection progresses, there is a gradual drop in CD4 lymphocytes, which causes the appearance of infections that may have atypical presentations [38], such as the condition presented by this patient. Furthermore, certain symptoms, particularly hemiparesis, have been reported in cases of patients co-infected with NCC and HIV [34], suggesting a possible link between the co-infection and the atypical presentation of NCC, as observed in this case. It is important to emphasize that HIV is not a criterion in the recommendations for diagnosing and treating NCC [36]; however, patients with AIDS residing in NCC-endemic areas deserve special attention for possible infection diagnosis. The management should be personalized, and regular clinical and imaging follow-ups are fundamental [32].

The described clinical case exemplifies a presentation of extraparenchymal neurocysticercosis, specifically involving the subarachnoid space. This form of NCC is considered more severe than its parenchymal counterpart, with a correspondingly unfavorable prognosis [20]. In general, the subarachnoid type can result in permanent hydrocephalus, entrapment of intracranial nerves, or cerebrovascular complications [39]. Often, when the inflammatory exudate compresses the intracranial nerve, it can lead to focal neurological deficits. If the oculomotor nerves are affected, it can result in extraocular muscle paralysis and double vision (diplopia). Compression of both the optic nerve and optic chiasm by the exudate can cause decreased vision and defects in the visual field [40]. This is clearly demonstrated by the patient’s clinical manifestations and the intricate nature of the treatment undertaken in this case. The management demanded a collaborative effort involving multiple medical disciplines, notably neurosurgery, to achieve the best possible clinical outcome. Moreover, postsurgical follow-up played a vital role, considering the intensity of the infection and the invasive procedures involved.

The treatment for neurocysticercosis can be either medical or surgical [41]. The main medical approach involves administering antiparasitic drugs, particularly praziquantel and albendazole, in addition to corticosteroids like dexamethasone to reduce inflammation [39]. In this particular case, the patient received albendazole [41], along with dexamethasone. According to the guidelines from the Infectious Diseases Society of America (IDSA) and the American Society of Tropical Medicine and Hygiene (ASTMH), the recommended treatment for patients with one to two viable parenchymal cysticerci is albendazole monotherapy for 10–14 days. On the other hand, it is only suggested to combine albendazole with praziquantel for 10–14 days in cases where patients have more than two viable parenchymal cysticerci, which was not the situation in this particular instance [42]. Furthermore, the patient presented with hydrocephalus, demanding the placement of a ventriculoperitoneal (VP) shunt, a procedure often required in subarachnoid neurocysticercosis due to persistent hydrocephalus, which resulted in a satisfactory clinical improvement. The VP shunt aims to alleviate elevated intracranial pressure and reduce mortality [39]. Consequently, adequate diagnosis and treatment are essential, mainly due to the small number of known cases and published research, leading to better long-term results and the prevention of complications, thus promoting a better quality of life for co-infected patients.

## 4. Conclusions

Neurocysticercosis (NCC) can exhibit a wide array of symptoms and clinical manifestations. In this case report, the patient presented with headaches, focal deficits, and hydrocephalus, yet interestingly, no seizure episodes were reported, despite their prevalence in many NCC cases. As a result, the diagnostic process involved multiple steps, wherein a comprehensive assessment of the medical history, laboratory tests, and imaging played a decisive role in devising the most appropriate intervention and therapeutic plan for the patient. It is important to underscore that this case represents a challenging instance of subarachnoid neurocysticercosis, necessitating a sophisticated and often multidisciplinary approach. The treatment encompassed surgical procedures and the management of potential complications, both of which were indispensable in saving the patient’s life.

## Figures and Tables

**Table 1:** Biochemical and Immunological Analysis of Cerebrospinal Fluid of the Patient.

Analyte	22/09/2022	26/09/2022	05/10/2022	17/03/2023	21/03/2023	26/03/2023	Reference Values
Glucose	18.0 mg/dL	27.0 mg/dL	29.0 mg/dL	44 mg/dL	28 mg/dL	61 mg/dL	44–66 mg/dL
pH	8	7.5	7.4	8.0	7.5	-	7.35–7.45
Protein	230.0 mg/dL	198.0 mg/dL	229.0 mg/dL	79.0 mg/dL	0.9 mg/dL	43 mg/dL	10–45 mg/dL
Lactate	20.8 mg/dL	19.1 mg/dL	19.0 mg/dL	25.8 mg/dL	28.3 mg/dL	19.5 mg/dL	9–19 mg/dL
LDH	41 UI/L	45 UI/L	45 UI/L	128 UI/L	226 UI/L	37 UI/L	1–35 UI/L
VDRL	Non-reactive	Non-reactive	Non-reactive	Non-reactive	Non-reactive	-	-
CRP	-	-	0.32 IU/L	0.90 IU/L	10.03 IU/L	0.12 IU/L	0–0.8 IU/L

Underlined values are outside the reference values, according to the laboratory of the General Hospital of Roraima (HGR).

**Table 2:** Physical, Chemical, and Cytological Analysis of Cerebrospinal Fluid.

Analyte	22/09/2022	26/09/2022	05/10/2022	17/03/2023	21/03/2023	26/03/2023	Reference Values
Coagulation	Absent	Absent	Absent	Absent	Absent	Absent	Absent
Sample color	Colorless	Yellowish	Colorless	Colorless	Colorless	Colorless	Colorless
Global leukocyte count	10	04	38	00	237	01	0–10 cells/mm^3^
Global erytrocyte count	00	01	02	500	130	250	0–0 cells/mm^3^
Polymorphonuclear leukocyte percentage	04%	-	04%	-	47%	-	2–10%
Mononuclear leukocyte percentage	96%	-	96%	-	53%	-	90–98%

Underlined values are outside the reference values, according to the laboratory of the General Hospital of Roraima (HGR).

## Data Availability

Data available on request from the authors.
